# Long-Term Clinical Efficacy of the Disc-FX Procedure in Contained Disc Herniation: A 7-Year Follow-Up from a Single-Center Cohort Study

**DOI:** 10.3390/jcm14186378

**Published:** 2025-09-10

**Authors:** Magdalena Rybaczek, Kacper Prokop, Karol Sawicki, Robert Rutkowski, Aleksander Lebejko, Grzegorz Perestret, Zenon Mariak, Paweł Grabala, Tomasz Łysoń

**Affiliations:** Department of Neurosurgery, Medical University of Bialystok, M. Sklodowskiej-Curie 24A, 15-276 Bialystok, Poland; prokop.kacper@o2.pl (K.P.); kar.saw89@gmail.com (K.S.); robert.rutkowski@umb.edu.pl (R.R.); alebejko@gmail.com (A.L.); grzegorz.perestret@gmail.com (G.P.); zenon.mariak@umb.edu.pl (Z.M.); pgrabala@wp.pl (P.G.); tomasz.lyson@umb.edu.pl (T.Ł.)

**Keywords:** Disc-FX, contained disc herniation, minimally invasive spine surgery, percutaneous, nucleotomy, nucleoplasty

## Abstract

**Background:** Contained lumbar disc herniation is a prevalent cause of chronic low back pain and functional impairment. The Disc-FX system, a minimally invasive, percutaneous technique integrating nucleotomy, nucleus ablation, and annuloplasty, offers a multimodal approach to managing early degenerative disc disease. Despite promising short-term outcomes, evidence regarding long-term effectiveness remains limited. **Methods:** This single-center cohort study evaluated 197 patients (median age: 48 years; 56.85% female) who underwent the Disc-FX procedure between 2017 and 2024. Patients were followed for up to 84 months. Pain and disability were assessed using a Visual Analog Scale (VAS) and the Oswestry Disability Index (ODI), respectively, while satisfaction was measured by the MacNab criteria. Multivariable models, including cumulative link models and linear mixed-effects models, were used to identify predictors of outcomes. **Results:** The Disc-FX procedure resulted in significant and sustained improvements in pain and function. Mean VAS scores decreased from 7.79 preoperatively to 4.31 at 12 months and remained below baseline at 84 months (5.05). ODI scores improved from 15.43 preoperatively to 9.62 at 36 months, rising slightly to 12.75 at 84 months. Good or excellent outcomes were reported in 66.9% of patients according to MacNab criteria. Male sex (OR = 0.41), longer symptom duration (OR = 0.85), and presence of radicular symptoms (OR = 0.39) were significantly associated with less favorable outcomes. Reoperation occurred in 26.4% of cases, predominantly within the first year and most frequently at L4/L5. Complications were rare (3.08%). **Conclusions:** This study provides robust evidence supporting the long-term clinical efficacy of the Disc-FX procedure in selected patients with contained lumbar disc herniation. While overall outcomes are favorable, optimal results depend on early intervention and careful patient selection, particularly in relation to symptom chronicity and the presence of radicular signs.

## 1. Introduction

Lumbar degenerative disc disease (DDD) is a prevalent spinal disorder and a leading cause of functional disability worldwide [1]. It typically presents as low back pain, often accompanied by radicular symptoms resulting from nerve root irritation or compression. While many patients achieve clinical improvement with conservative treatments, some individuals continue to experience persistent and disabling complaints that significantly interfere with daily activities. Notably, magnetic resonance imaging (MRI) examination in many cases reveals only subtle, contained disc herniations, occasionally associated with annular tears presenting as high-intensity zones (HIZs) on T2-weighted images—findings that insufficiently explain the intensity of clinical symptoms [2]. Nevertheless, even early degenerative changes in the lumbar spine may elicit considerable pain and functional impairment.

Recent innovations in spinal multimodal approaches have facilitated the early detection and management of intervertebral disc degeneration, potentially delaying the progression to more advanced structural deterioration [3].

A rational treatment strategy emphasizes the use of the least invasive options, particularly for patients who are not ideal candidates for conventional surgery due to mild or inconclusive imaging findings.

Within this minimally invasive paradigm, three percutaneous, non-endoscopic spinal techniques have gained particular clinical relevance—nucleotomy, nucleoplasty, and annuloplasty [4]. Technological advances in spine surgery enabled the integration of these key features into a single procedure—the Disc-FX system—which combines manual disc decompression, radiofrequency modulation of the nucleus pulposus, and thermal ablation of nociceptive fibers within the annulus fibrosus.

This comprehensive approach has shown promising results, as several clinical studies have reported significant reductions in pain, improved functionality, and a low incidence of complications [5,6,7]. However, the majority of published data are based on short- to mid-term follow-up, leaving the long-term durability and clinical trajectory of outcomes insufficiently characterized [7]. Moreover, appropriate patient selection remains a critical factor in optimizing outcomes. These limitations underscore the importance of long-term clinical observation to clarify the durability of clinical benefits.

To address these gaps, the present study aimed to conduct a long-term evaluation of the Disc-FX procedure in patients with early-stage degenerative lumbar disc disease, focusing on pain intensity, functional status, and patient-reported satisfaction over a 7-year follow-up period.

## 2. Materials and Methods

### 2.1. Characteristics of the Study Sample

This study analyzed data from a cohort of 197 adult patients who underwent the Disc-FX procedure between 2017 and 2024. All patients presented with symptoms of lumbalgia and/or radiculopathy and had preoperative magnetic resonance imaging (MRI) confirming the presence of a contained lumbar disc herniation, defined according to the nomenclature and classification proposed by Appel et al. [8] as a focal protrusion or contained extrusion without rupture of the annulus fibrosus and sequestration of the nucleus pulposus. Exclusion criteria included: (1) sequestered or migrated fragments, (2) severe spinal canal or foraminal stenosis, (3) spinal instability or spondylolisthesis, (4) previous surgery at the index level, and (5) systemic contraindications to minimally invasive surgery. These strict selection criteria were applied to ensure the homogeneity of the study cohort and to optimize postoperative outcomes.

The sample comprised both female and male participants, with 112 females (56.85%) and 85 males (43.15%), resulting in a female-to-male ratio of 1.32:1. The majority of patients were middle-aged adults, with a median age of 48 years (*IQR*: 38–57 years) and an overall age range of 18 to 76 years. The median body mass index (BMI) of the cohort was 27.38 kg/m^2^ (*IQR*: 24.38–30.06 kg/m^2^), classifying the group, on average, with BMI values ranging from 17.3 to 38.06 kg/m^2^.

### 2.2. Methods

Pain intensity was measured using a Visual Analog Scale (VAS), which consists of a 10 cm horizontal line. The line is marked with “0 = no pain” at the left end and “10 = worst imaginable pain” at the right end. Patients were instructed to indicate their perceived level of pain by marking a point on the line.

All patients were assessed using the Oswestry Disability Index (ODI), designed to assess coping with daily activities. The ODI consists of 10 sections, each scored from 0 to 5, with a total possible score ranging from 0 to 50 points. In this study, raw scores (0–50) were used without conversion to percentage values. Higher scores indicate a greater degree of disability.

Patient satisfaction with the surgical outcome was evaluated using the MacNab criteria. It is a four-level scale used to evaluate postoperative outcomes based on pain relief and functional recovery, reflecting the patient’s subjective sense of satisfaction with the surgical result. The classification depends on the patient’s ability to return to daily and occupational activities with or without residual symptoms [9]. According to this scale, postoperative results are classified as: 5—*Excellent*—no pain and return to normal work and activities; 4—*Good*—occasional non-radicular pain with the ability to return to modified work; 3—*Fair*—some improvement but persistent limitations due to intermittent pain; 2—*Poor*—no improvement, and 1—*Failure*—worsening of symptoms. These data were collected during the final follow-up visit 7 years after the procedure by a surgeon who had not performed the procedures and who was not involved in obtaining other clinical data such as the VAS or ODI scores. This was a deliberate methodological decision, designed to avoid introducing bias and to ensure that patients were able to openly express their honest opinions without any perceived pressure or expectations from the operating surgeon.

Clinical data included demographics, type of work (sedentary, physical, or retired), smoking status, symptom duration, surgical level, laterality of intervention, preoperative and postoperative treatments, complications, and reoperation history. Symptom assessment involved the presence of lumbalgia and radicular symptoms, such as sensory or motor deficits. Follow-up data on pain and functional status were collected at multiple time points up to 7 years (84 months) postoperatively.

### 2.3. Statistical Analysis

In the conducted study, a significance level of α = 0.05 was adopted, indicating an acceptance of a 5% risk of committing a Type I error. The intervention outcomes were presented as the mean (M) and standard deviation (SD) to highlight the variance in the case of the ordinal variable (McNab score) and the variability of parameters over time (VAS, ODI scores). For the rest of the continuous variables, the median (*Mdn*) was used as the measure of central tendency due to its robustness against outliers. Additionally, to better illustrate the data distribution, the first (*Q1*) and third quartiles (*Q3*) were reported, collectively defining the interquartile range (*IQR*), which encompasses 50% of the observations in the sample. For categorical variables, the number of observations (*n*) and the percentage distribution of individual categories were provided.

The identification of baseline parameters that significantly predict the McNab score among patients undergoing the minimally invasive Disc FX procedure was performed using a multivariable approach. A cumulative link model (CLM) with a logit link function was employed to analyze the ordinal outcome of the McNab score. Continuous predictors were centered around their respective medians to improve model interpretability and reduce multicollinearity. Odds ratios (*OR*s) with 95% confidence intervals (*95% CIs*) were calculated for each predictor to quantify the strength and direction of their association with the McNab score.

The performance of the CLM was validated using several appropriate goodness-of-fit (GOF) procedures to ensure the model’s reliability and predictive accuracy. The proportional odds assumption, a fundamental requirement of the CLM, was tested using the Brant test. In addition to the Brant test, other key goodness-of-fit tests were applied. These included the Hosmer–Lemeshow test [10,11,12] and the Lipsitz test [13]. To provide further insight into the predictive accuracy of the model, prediction errors were evaluated using error or loss functions, such as the Brier score. Summary measures of the model’s predictive strength were derived using Ugba and Gertheiss’s framework.

To address concerns about multicollinearity, the Variance Inflation Factor (VIF) was calculated for all predictors for each model. A threshold of VIF < 3.0 was used to indicate the absence of significant bias due to multicollinearity.

The effects of selected covariates on VAS and ODI outcomes for longitudinal data were modeled using a linear mixed-effects model (LMM) with maximum likelihood estimation, where the outcome variable is measured repeatedly over time for each patient. The model specified in Appendix A accounts for both fixed effects (population-level effects) and random effects (individual-level variability). It includes a nonlinear effect of time using natural cubic splines, various patient-level covariates, and a random slope for time to capture individual variability in the trajectory of the outcome. This approach enables a robust and flexible analysis of longitudinal data, even when follow-up periods are uneven across participants.

### 2.4. Characteristics of the Statistical Tool

Analyses were conducted using the R statistical language (version 4.3.3;) [14] on Windows 11 pro 64 bit (build 22,631), using the packages *lme4* (version 1.1.35.2); [15], *Matrix* (version 1.6.5; [16]), *ordinal* (version 2023.12.4.1; [17]), *sjPlot* (version 2.8.15; [18]), *performance* (version 0.12.3; [19]), *report* (version 0.5.8; [18]), *gtsummary* (version 1.7.2; [20]), *gofcat* (version 0.1.2; [21]), *ggplot2* (version 3.5.0; [22]), and dplyr (version 1.1.4; [23]).

### 2.5. Surgical Procedure

**Figure 1:**
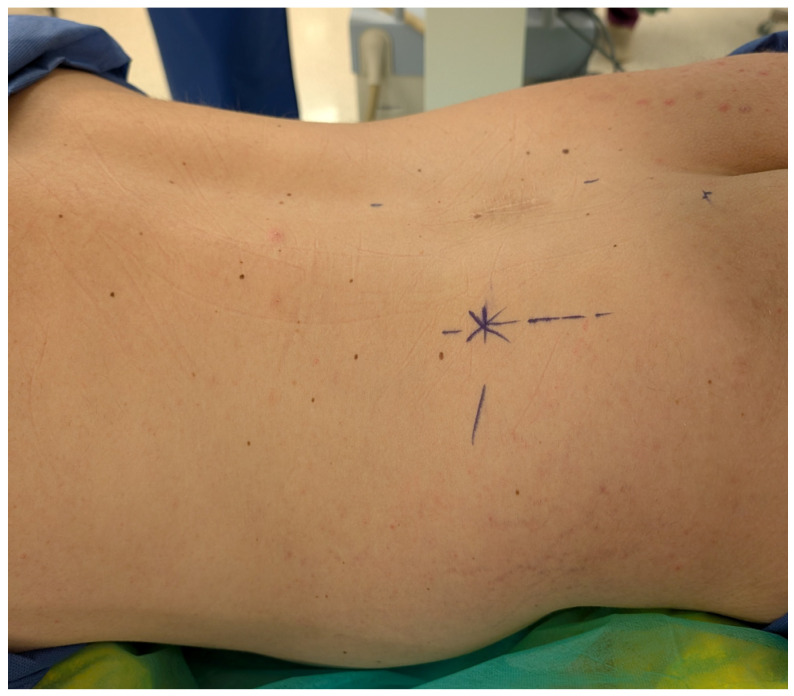
Patient positioning in prone posture for the Disc-FX procedure. Optimal positioning allows fluoroscopic access to the lumbar spine under local anesthesia and conscious sedation.

**Figure 2:**
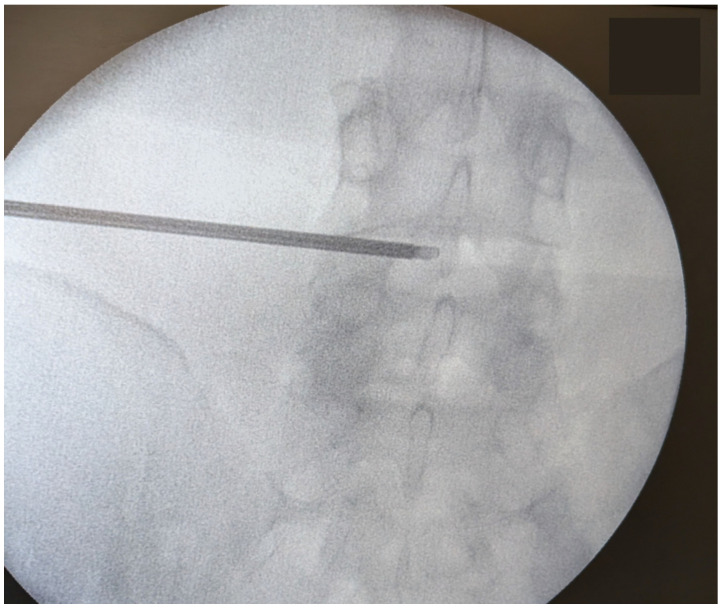
Fluoroscopic guidance for needle insertion into the affected disc space.

The procedure is carried out in three consecutive stages: nucleus decompression, nucleus ablation, and modulation of the annulus. Patients are placed in a prone position to allow optimal access to the lumbar spine (Figure 1). It is performed under local anesthesia with conscious sedation, enabling real-time feedback to reduce the risk of nerve injury. Using fluoroscopic guidance, a needle is inserted percutaneously into the affected disc space (Figure 2).

**Figure 3:**
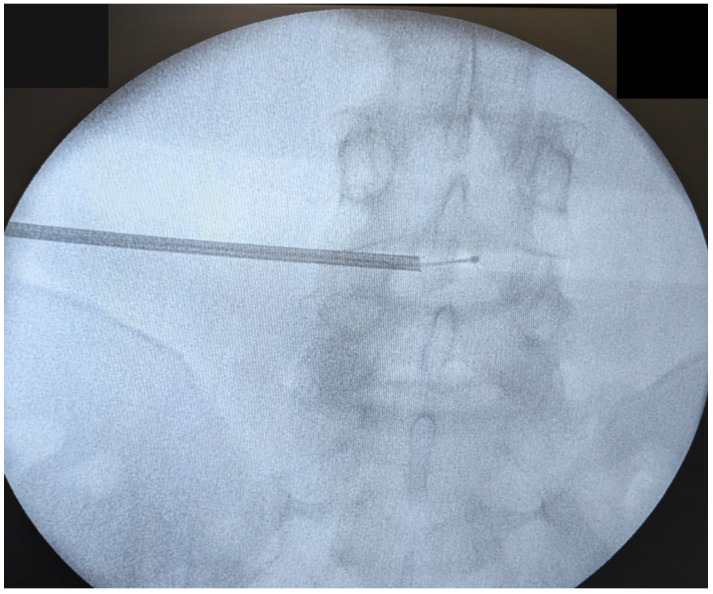
Electrode positioning at multiple angles under fluoroscopic guidance facilitates uniform collagen contraction and pressure reduction.

After accurate place confirmation on anteroposterior and lateral X-ray views, a guidewire and dilators are used to introduce a tubular system for manual decompression. The next step is nucleus ablation with a radiofrequency electrode, followed by annular modulation, which shrinks collagen fibers and reduces intradiscal pressure (Figure 3).

**Figure 4:**
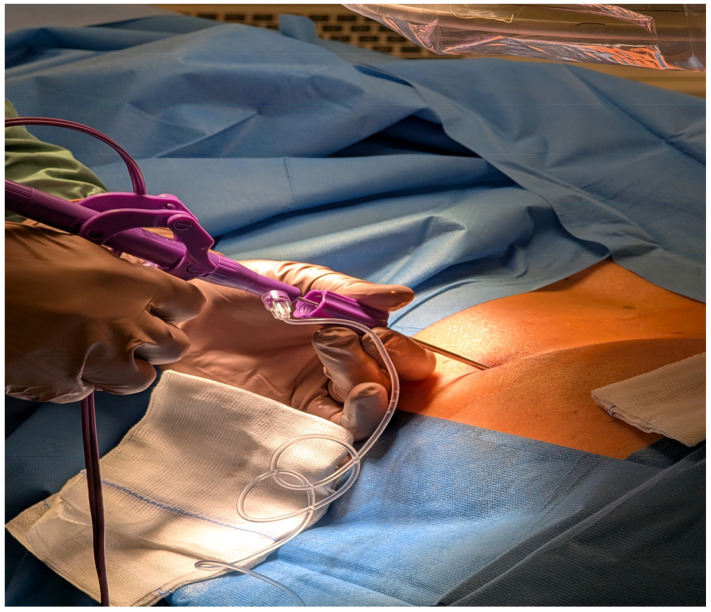
Radiofrequency annuloplasty. Thermal coagulation is applied to annular tears and nociceptive fibers to promote stabilization and symptom relief.

The final stage, annuloplasty, involves coagulation of annular tears and nociceptive fibers, applying controlled radiofrequency sweeps to stabilize the disc and prevent further nuclear migration (Figure 4). The procedure lasts approximately 15–20 min and concludes with device withdrawal and skin closure with Steri-Strips.

## 3. Results

### 3.1. Characteristics of Clinical Pre- and Postoperative Parameters

According to the results in Table 1, smoking was reported in 36.55% of patients. The majority of patients were engaged in sedentary work (52.79%), while 29.44% performed physical labor and 17.77% were retirees.

The median symptom duration was 3 years. Lumbalgia was reported in 82.23% of patients, and 74.62% presented with root-related symptoms, such as sensory or motor deficits. Left-sided interventions accounted for 50.25% of cases, while right-sided interventions represented 49.75%.

Most procedures were performed at L4/L5 (81.73%), followed by L5/S1 (7.11%) and L3/L4 (9.14%).

Preoperative rehabilitation was utilized by only 28.93% of patients, while postoperative rehabilitation increased to 37.56%. Steroid blocks were administered in 4.06% of patients preoperatively and in 25.38% postoperatively, likely reflecting their use as adjunctive therapy for residual or recurrent symptoms.

Reoperations were required in 26.40% of patients, with most undergoing a single re-intervention (75%). The median time to the first re-intervention was 12 months, indicating that residual or recurrent symptoms tend to manifest within the first year after surgery. Notably, 76.92% of reoperations were performed at the L4/L5 level, mirroring the predominance of this level as the primary intervention site.

Complications were rare, occurring in only 3.08% of cases, which underscores the safety of the Disc FX procedure.

### 3.2. Characteristic of the Clinical Outcomes

#### 3.2.1. MacNab Score

**Figure 5:**
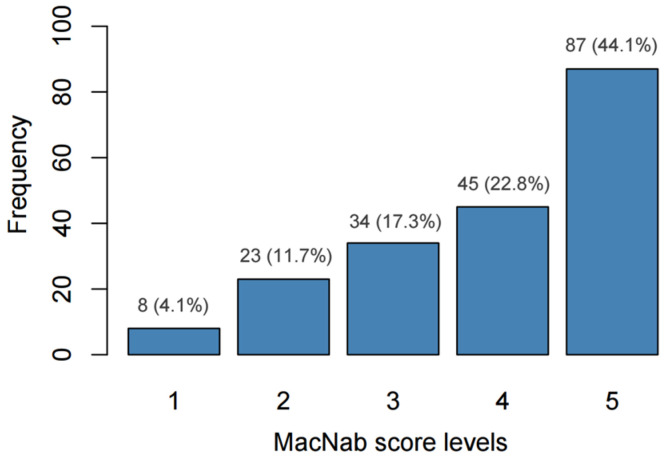
Distribution of MacNab score levels indicating postoperative outcomes in the studied cohort.

Analysis of MacNab scores (Figure 5) showed that 87 patients (44.1%) achieved level 5 and 45 patients (22.8%) reached level 4. Lower outcome levels were less common, with 34 patients (17.3%) at level 3, 23 patients (11.7%) at level 2, and 8 patients (4.1%) at level 1 (Figure 5).

#### 3.2.2. Visual Analog Scale (VAS)

**Figure 6:**
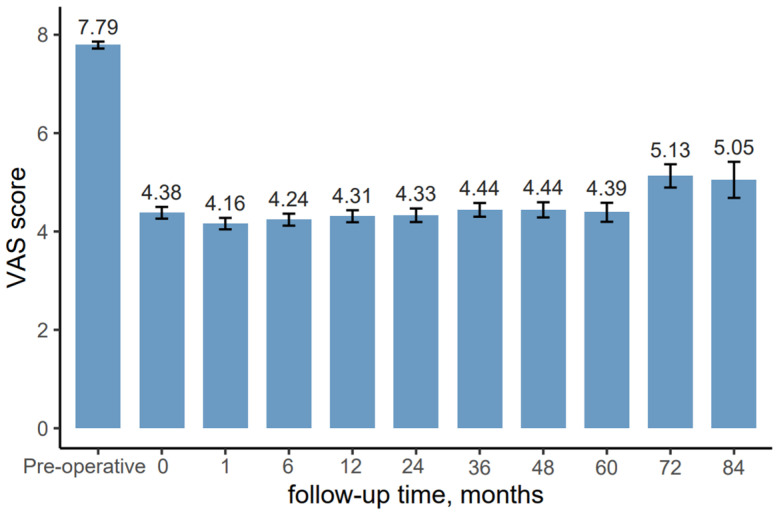
Mean VAS scores with standard errors for patients before and after Disc FX procedure.

The percutaneous Disc FX technique demonstrates significant efficacy in reducing pain as measured by the Visual Analog Scale (Figure 6). At baseline, patients reported severe pain with a mean VAS score of 7.79 (±0.99). Following the procedure, there was a substantial immediate reduction in pain, with the mean VAS score decreasing to 4.38 (±1.67).

Over the first postoperative year, the pain reduction was sustained, with VAS scores remaining stable between 4.16 (±1.63) at 1 month and 4.31 (±1.71) at 12 months. Beyond the first year, the mean VAS scores from 24 to 60 months demonstrated continued stability, ranging between 4.33 (±1.63) at 24 months and 4.39 (±1.78) at 60 months.

However, after the 6-year mark, a gradual increase in pain levels was observed. The mean VAS score rose to 5.13 (±1.74) at 72 months and slightly decreased to 5.05 (±1.64) at 84 months.

It is important to note that the sample size decreased over the follow-up period, with 197 patients at baseline and only 20 patients remaining at the 84-month follow-up.

#### 3.2.3. ODI Score

**Figure 7:**
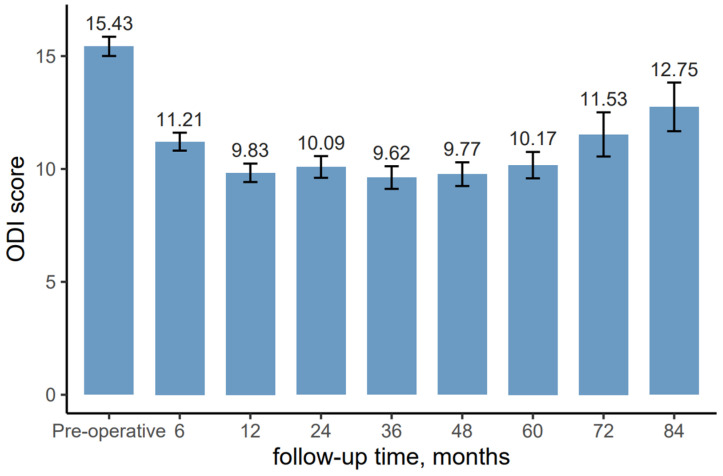
Mean ODI scores with standard errors for patients before and after Disc FX procedure.

Before surgery, the mean ODI score was 15.43 (±5.98), reflecting a significant level of functional disability related to the patient’s condition (Figure 7). By 6 months postoperatively, the ODI score decreased to 11.21 (±5.59).

Over the subsequent follow-up periods, the functional improvement persisted, with the ODI score reaching its lowest point at 36 months, with a mean of 9.62 (±5.60). Between 12 and 60 months, the ODI scores fluctuated slightly with values ranging from 9.83 (±5.73) at 12 months to 10.17 (±5.41) at 60 months.

After 6 years, a gradual increase in ODI scores was observed, with scores rising to 11.53 (±5.70) at 72 months and 12.75 (±4.82) at 84 months.

### 3.3. Estimation of the Effect of the Baseline Parameters on the McNab Score Among Patients Undergoing the Minimally Invasive Disc FX Procedure

The predictor variables included nine potential predictors, specifically, demographic characteristics (e.g., sex, age, BMI), lifestyle factors (e.g., smoking, work types), and clinical features (e.g., duration of ailment, presence of lumbalgia, root symptoms).

The results of the fitted CLM model reported in Table 2 suggested that male sex, longer symptom duration, and the presence of root symptoms were associated with worse McNab scores (thus can be characterized as risk factors). In contrast, factors such as age, BMI, smoking status, and occupational type did not have a significant effect on the outcome.

Specifically, males had significantly lower odds ratios of achieving higher McNab scores compared to females, with an odds ratio of 0.41 (*95% CI*: 0.22–0.76, *p* = 0.005). This statistically significant result suggests that female patients may have a higher likelihood of better surgical outcomes.

In contrast, age did not show a significant association with the McNab score (*OR*: 1.02, *95% CI*: 0.99–1.05, *p* = 0.198). Similarly, BMI centered at a median of 27.38 kg/m^2^ was also not a significant predictor (*OR*: 1.02, *95% CI*: 0.95–1.09, *p* = 0.613).

Smoking, another factor often implicated in poorer surgical outcomes, also did not significantly influence the McNab score (*OR*: 0.95, *95% CI*: 0.53–1.71, *p* = 0.861). When examining the type of work performed, neither physical labor nor sedentary work showed statistically significant associations with the McNab score. For physical laborers, the *OR* = 1.10 (*95% CI*: 0.38–3.21, *p* = 0.857), while for sedentary workers, the *OR* = 0.54 (*95% CI*: 0.21–1.40, *p* = 0.208). One of the most significant predictors was the duration of symptoms before surgery, centered at a median of 3 years. Longer symptom duration was associated with worse outcomes, with an *OR* = 0.85 (*95% CI*: 0.73–1.00, *p* = 0.045). The presence of root symptoms—such as radicular pain, numbness, or weakness—was another statistically significant predictor of lower McNab scores (*OR*: 0.39, *95% CI*: 0.20–0.76, *p* = 0.006).

Moreover, lumbalgia (isolated back pain) did not significantly impact the McNab score (*OR*: 0.55, *95% CI*: 0.27–1.15, *p* = 0.111).

### 3.4. Estimation of the Effect of the Clinical Parameters on the VAS Score Among Patients Undergoing the Minimally Invasive Disc FX Procedure

Table 3 presents the results of a linear mixed-effects model (LMM) examining the relationship between selected covariates and VAS (Visual Analog Scale) scores. The covariates included in the model are sex, age, BMI, past treatments before fracture (FX), surgery after fracture, steroid blocks after the intervention, steroid blocks before the intervention, rehabilitation after surgery, rehabilitation before surgery, and complications. These variables represent a combination of demographic, clinical, and treatment-related factors that may influence the trajectory of pain or functional outcomes, as measured by VAS scores, which serve as the outcome.

The model includes 199 individuals (Nid) with a total of 1499 observations (Nobs). The marginal R^2^ for the model is 0.217, indicating that approximately 21.7% of the variance in VAS scores is explained by the fixed effects included in the model. The analysis incorporates a combination of time-related nonlinear effects and a wide range of patient-level covariates, such as demographic and clinical characteristics, as well as treatment-related variables.

The intercept of the model, representing the average VAS score when all predictors are at their reference or centered values, is estimated at 5.10 (*95% CI*: 4.89–5.30, *p* < 0.001). This reflects the baseline level of pain or functional impairment for the reference group.

The time-related predictors reveal a highly nonlinear trajectory of VAS scores over time. While the linear term for time is not statistically significant (β = −0.42, *p* = 0.155), the second-and third-degree terms are highly substantial (β = −1.36 and β = −2.80, both *p* < 0.001), indicating that the outcome follows a pronounced curvilinear pattern with a steep decline in VAS scores as time progresses. The fourth-degree term, while approaching significance (β = 0.72, *p* = 0.089), suggests a possible subtle reversal in the trajectory at later stages, though this remains uncertain.

Sex, age, and BMI show no significant associations with VAS scores, suggesting that these demographic and anthropometric factors at baseline do not significantly influence the outcome in this population. Similarly, prior treatments before the fracture, steroid blocks after the intervention, and rehabilitation before surgery are not significantly associated with VAS scores, indicating that these factors do not meaningfully impact the trajectory of pain or functionality.

Surgery after the fracture is associated with a significant increase in VAS scores (β = 1.10, *95% CI*: 0.86–1.33, *p* < 0.001), suggesting that individuals who underwent surgery experienced higher pain or functional impairment compared to those who did not.

Moreover, steroid blocks before the intervention are also significantly associated with higher VAS scores (β = 0.62, *95% CI*: 0.08–1.17, *p* = 0.024), indicating that pre-intervention steroid use may be a marker of more severe baseline conditions or persistent pain. Rehabilitation after surgery shows a positive association with VAS scores (β = 0.36, *95% CI*: 0.15–0.57, *p* = 0.001), which may reflect the fact that patients requiring post-surgical rehabilitation tend to have more severe cases or slower recovery trajectories. Complications are significantly associated with an increase in VAS scores (β = 0.64, *95% CI*: 0.07–1.21, *p* = 0.027), underscoring the negative impact of adverse events on patient outcomes.

### 3.5. Estimation of the Effect of the Clinical Parameters on the ODI Score Among Patients Undergoing the Minimally Invasive Disc FX Procedure

Table 4 presents the results of a linear mixed-effects model (LMM) examining the relationship between the selected covariates (the list of covariates was the same as in Section 2.4) and ODI scores as outcome. The marginal *R*^2^ = 0.382, indicating that 38.2% of the variance in ODI scores is explained by the fixed effects, while the conditional *R*^2^ = 0.879, showing that 87.9% of the variance is explained when both fixed and random effects are considered. These results highlight the importance of accounting for individual variability in the model. The intercept represents the baseline level of disability in the study sample, estimated at 13.14 (*95% CI*: 12.00–14.29, *p* < 0.001).

The nonlinear time effects in the regression model reveal a complex trajectory in ODI scores over time. The significant first-degree term (β = −5.05, *p* < 0.001) reflects a steep initial reduction in disability during the early recovery period, with the second-degree (β = −6.28, *p* < 0.001) and third-degree (β = −13.21, *p* < 0.001) terms indicating a continued but nonlinear decline in ODI scores during the intermediate phases. These results show that much of the improvement occurs early, with the rate of recovery accelerating further into the intermediate stages before slowing.

The nonsignificant fourth-degree term (β = −1.05, *p* = 0.135) suggests that after the initial decline and stabilization, there is a transition to a phase where further reductions in ODI scores are negligible. However, the nonlinear nature of the time effects implies that this stabilization is not permanent, as the modeled trajectory also accommodates the possibility of a late-phase increase in ODI scores, indicative of a gradual deterioration in disability. The potential late-phase deterioration underscores the need for long-term follow-up and interventions to address the factors that contribute to worsening functional outcomes after the initial recovery period.

Surgery after the fracture is significantly associated with higher ODI scores (β = 4.41, *p* < 0.001), indicating worse functional outcomes for patients who underwent surgery, likely reflecting the severity of cases requiring surgical intervention. Complications also show a strong association with higher ODI scores (β = 6.73, *p* < 0.001), underscoring the negative impact of adverse events on recovery and long-term functionality. Rehabilitation after surgery is associated with higher ODI scores (β = 1.64, *p* = 0.019), which may reflect the greater disability of patients who require rehabilitation. Although pre-intervention steroid blocks approach significance (β = 3.04, *p* = 0.084), this suggests a potential trend toward worse disability outcomes in patients receiving these treatments, possibly reflecting more severe baseline conditions.

## 4. Discussion

### 4.1. Clinical Effectiveness and Patient Satisfaction over Time

This study presents one of the longest follow-up evaluations of the Disc-FX technique, offering valuable insights into its long-term clinical utility for patients with early-stage degenerative lumbar disc disease. The results confirm the sustained efficacy of the procedure, with marked improvement in pain, function, and patient satisfaction, particularly in the first years after surgery.

These results are consistent with the systematic review conducted by Panagopoulos et al. [24], who reported favorable outcomes in 87.6% of patients. However, most studies included in the review had follow-up durations of 24 months or less, which highlights the added value of our extended observation period.

The mean reduction in pain intensity (VAS) from 7.8 at baseline to 4.3 at 12 months presented in our analysis corresponds to an absolute change of 3.5 points, which exceeds the commonly accepted minimal clinically important difference (MCID) of 1.5–2.0 points for a 0–10 pain scale [25,26]. These results are comparable to, or slightly less pronounced than, those reported after standard open procedures such as microdiscectomy or laminectomy in patients with larger and sequestered disc herniations, where VAS reductions of 4–5 points are typically observed [27,28]. Nevertheless, the magnitude of pain relief achieved in our cohort aligns well with previously published results for percutaneous techniques, including Disc-FX, in patients with contained herniations [5,6,7,24].

Concerning functional outcomes, the mean ODI score improved from 15.4 at baseline to 9.6 at 36 months, representing a raw reduction of 5.8 points. When expressed on a 100-point scale, this corresponds to an improvement of approximately 11.6 percentage points. Although the baseline ODI values in our cohort were relatively low—reflecting that only patients with contained herniations and moderate disability were enrolled—this improvement still exceeds the generally accepted MCID threshold for the ODI, which is approximately 10 percentage points [29,30]. Therefore, despite the moderate baseline disability, these findings indicate a clinically meaningful long-term functional benefit.

### 4.2. Factors Associated with Poorer Pain and Functional Outcomes

Multivariate analysis identified male sex, longer symptom duration, and radicular symptoms as independent predictors of poorer surgical outcomes. The reduced likelihood of favorable results in men, consistent with previous findings [31], may be related to differences in pain perception, psychosocial factors, rehabilitation adherence, or biological mechanisms such as the neuroprotective and anti-inflammatory effects of estrogen [32].

Prolonged symptom duration was linked to poorer outcomes, highlighting the importance of timely surgery when conservative treatment fails. This may reflect irreversible neural changes [32,33] as well as psychosocial consequences of chronic pain that hinder recovery and quality of life [34].

Radicular symptoms were a strong negative predictor of outcome, reflecting the difficulty of treating nerve-related pathology, as prolonged or severe root involvement may lead to persistent neuropathic pain or motor deficits even after adequate decompression [35,36].

Preoperative steroid injections were associated with higher postoperative VAS scores and a trend toward worse ODI, likely reflecting more severe baseline pathology rather than a direct adverse effect [27]. Similarly, the link between postoperative rehabilitation and poorer outcomes probably reflects reverse causality, as patients with greater symptom severity or slower recovery were more likely to undergo rehabilitation. The heterogeneous nature of “rehabilitation” limits the interpretability of this variable in our study, as specific modalities and timing were not distinguished. Future research should stratify rehabilitation approaches to assess their impact on the recovery process [37].

### 4.3. Factors Not Significantly Associated with Outcomes

In our cohort, age, sex, BMI, smoking status, occupational type, operated level, and duration of symptoms were not significantly associated with postoperative outcomes. Similarly, outcomes did not differ between patients with isolated low back pain and those with radicular symptoms. These negative findings should be interpreted with caution due to the limited sample size and single-center design.

### 4.4. Segmental Vulnerability, Reoperation Patterns and Complications

In the present study, several variables were associated with worse postoperative pain and functional outcomes. These included reoperation, postoperative complications, and preoperative steroid injections.

Patients who required reoperation reported significantly higher levels of pain (β = 1.10) and disability (β = 4.41). This finding is consistent with previous studies, which have shown that revision spinal procedures are often associated with worse outcomes compared to primary surgeries [37,38]. Reoperations may indicate more complex or recurrent pathology, such as persistent disc degeneration, instability, or epidural fibrosis, all of which can compromise long-term surgical success.

Reoperations occurred in 26.4% of patients, most often within the first year, reflecting early recurrence or persistence of symptoms. Although the Disc-FX procedure aims to relieve pain and restore function, it does not arrest the underlying degenerative cascade. Intervertebral discs continue to undergo age-related deterioration, which can contribute to symptom recurrence or adjacent segment involvement [39].

The L4/L5 level was both the most frequently treated and reoperated segment, consistent with its biomechanical vulnerability at the lumbosacral junction [40].

Several factors may contribute to reoperation, including premature activity resumption, insufficient core stability, biomechanical imbalance, and individual variability in healing or adherence [41]. Repeat surgery thus reflects the progressive nature of disc disease rather than procedural failure. Moreover, the psychological burden of reoperation and postoperative complications—though infrequent—was strongly associated with worse pain and function, underscoring the importance of prevention and early management [42].

Postoperative complications, although infrequent in this cohort, were strongly associated with poorer outcomes—both in terms of pain (β = 0.64) and functional limitation (β = 6.73). Even minor complications, such as infection, hematoma, or transient neurological symptoms, can lead to extended recovery times, increased patient distress, and reduced satisfaction with treatment.

### 4.5. Limitations

This study has several limitations. While we used standardized instruments such as the MacNab criteria, the assessment of patient satisfaction was not stratified by follow-up intervals, which limits insight into temporal changes in perceived benefit. Additionally, postoperative rehabilitation was not categorized by modality, frequency, or intensity, which reduced the interpretability of its observed association with clinical outcomes.

## 5. Conclusions

This study represents one of the longest follow-up assessments of the Disc-FX procedure to date. The overall therapeutic outcomes are favorable, supporting its role as a viable treatment option, particularly among patients with contained lumbar disc herniation, who do not respond to prolonged conservative therapy and are not candidates for more invasive surgical interventions.

## Figures and Tables

**Table 1:** Characteristics of pre- and postoperative parameters.

Characteristic	N	n (%)
*Demographics and lifestyle factors*		
Smoking	197	72.00 (36.55%)
Type of work performed	197	
not applicable (retiree)		35 (17.77%)
physical		58 (29.44%)
sedentary		104 (52.79%)
*Clinical and symptom characteristics*		
Duration of the ailment (years)	197	3.00 (2.00, 4.00)
Symptoms of lumbalgia	197	162 (82.23%)
Root symptoms (sensation/force)	197	147 (74.62%)
Intervention details		
Side of intervention:	197	
left		99 (50.25%)
right		98 (49.75%)
Current operation level:		
Th12/L1	197	1 (0.51%)
L1/L2	197	3 (1.52%)
L2/L3	197	6 (3.05%)
L3/L4	197	18 (9.14%)
L4/L5	197	161 (81.73%)
L5/S1	197	14 (7.11%)
*Preoperative and postoperative treatments*		
Past treatments before FX	197	24 (12.18%)
Steroid blocks before the intervention	197	8 (4.06%)
Steroid blocks after intervention	197	50 (25.38%)

**Table 2:** Thresholds and coefficients of fitted CLM model, *n_obs_* = 197, *R*^2^*_Ugba (log)_* = 0.123.

Predictors	McNab Score		
	OR	95% CI	*p*
*Threshold coefficients*			
1|2	0.01	0.00–0.02	**<0.001**
2|3	0.03	0.01–0.10	**<0.001**
3|4	0.07	0.02–0.28	**<0.001**
4|5	0.20	0.05–0.74	**0.016**
*Regression coefficients*			
sex [female]	Reference category		
sex [male]	0.41	0.22–0.76	**0.005**
Age (centered by median 48.0 years)	1.02	0.99–1.05	0.198
BMI (centered by median 27.38 kg/m^2^)	1.02	0.95–1.09	0.613
smoking [no]	Reference category		
smoking [yes]	0.95	0.53–1.71	0.861
physical work [no]	Reference category		
physical work [yes]	1.10	0.38–3.21	0.857
sendentary work [no]	Reference category		
sendentary work [yes]	0.54	0.21–1.40	0.208
ailment duration (centered by median 3.0 years)	0.85	0.73–1.00	**0.045**
lumbalgia symptom [no]	Reference category		
lumbalgia symptom [yes]	0.55	0.27–1.15	0.111
root symptom [no]	Reference category		
root symptom [yes]	0.39	0.20–0.76	**0.006**

**Table 3:** LMM regression coefficients, *N_id_* = 199, *N_obs_* = 1499, *R*^2^*_marginal_* = 0.217.

Predictors	VAS Score		
	β	95% CI	*p*
(Intercept)	5.10	4.89–5.30	**<0.001**
time [1st degree]	−0.42	−1.00–0.16	0.155
time [2nd degree]	−1.36	−1.98–0.75	**<0.001**
time [3rd degree]	−2.80	−3.49–2.11	**<0.001**
time [4th degree]	0.72	−0.11–1.55	0.089
Sex [female]	Reference level		
Sex [male]	0.12	−0.08–0.32	0.248
Age (centered by median 48.0 years)	−0.00	−0.01–0.01	0.667
BMI (centered by median 27.38 kg/m^2^)	−0.01	−0.03–0.02	0.627
Past treatments before FX [no]	Reference level		
Past treatments before FX [yes]	−0.04	−0.36–0.27	0.793
Surgery after FX [no]	Reference level		
Surgery after FX [yes]	1.10	0.86–1.33	**<0.001**
Steroid blocks after intervention [no]	Reference level		
Steroid blocks after intervention [yes]	−0.00	−0.24–0.24	0.972
Steroid blocks before the intervention [no]	Reference level		
Steroid blocks before the intervention [yes]	0.62	0.08–1.17	**0.024**
Rehabilitation after surgery [no]	Reference level		
Rehabilitation after surgery [yes]	0.36	0.15–0.57	**0.001**
Rehabilitation before surgery [no]	Reference level		
Rehabilitation before surgery [yes]	−0.07	−0.30–0.16	0.565
Complications [no]	Reference level		
Complications [yes]	0.64	0.07–1.21	**0.027**

**Table 4:** Regression coefficients of fitted LMM model, *N_id_* = 195, *N_obs_* = 1089.

Predictors	ODI Score		
	β	95% CI	*p*
(Intercept)	13.14	12.00–14.29	**<0.001**
time [1st degree]	−5.05	−5.83–−4.27	<0.001
time [2nd degree]	−6.28	−7.26–−5.30	**<0.001**
time [3rd degree]	−13.21	−14.56–−11.85	**<0.001**
time [4th degree]	−1.05	−2.42–0.33	0.135
Sex [female]		Reference level	
Sex [male]	0.23	−1.08–1.54	0.727
Age (centered by median 48.0 years)	−0.00	−0.01–0.01	0.390
BMI (centered by median 27.38 kg/m^2^)	0.05	−0.11–0.22	0.521
Past treatments before FX [no]		Reference level	
Past treatments before FX [yes]	0.33	−1.66–2.33	0.742
Surgery after FX [no]		Reference level	
Surgery after FX [yes]	4.41	2.89–5.92	**<0.001**
Steroid blocks after intervention [no]		Reference level	
Steroid blocks after intervention [yes]	−0.39	−1.92–1.13	0.614
Steroid blocks before the intervention [no]		Reference level	
Steroid blocks before the intervention [yes]	3.04	−0.41–6.49	0.084
Rehabilitation after surgery [no]		Reference level	
Rehabilitation after surgery [yes]	1.64	0.27–3.01	**0.019**
Rehabilitation before surgery [no]		Reference level	
Rehabilitation before surgery [yes]	−1.21	−2.66–0.25	0.104
Complications [no]		Reference level	
Complications [yes]	6.73	2.98–10.48	**<0.001**

Note: *N_id_*—number of individuals; *N_obs_*—number of observations.

## Data Availability

Data are contained within the article.

## Abbreviations

DDD	degenerative disc disease
MR	magnetic resonance imaging
HIZs	high-intensity zones
BMI	median body mass index
VAS	Visual Analog Scale
ODI	Oswestry Disability Index
CLM	cumulative link model
ORs	odds ratios
CI	confidence intervals
GOF	goodness-of-fit
VIF	Variance Inflation Factor
LMM	linear mixed-effects model
MCID	minimal clinically important difference

## Appendix A. Longitudinal Model Specification

**Level 1: Within-patient model (repeated measures)**

At time *t* for patient *i*, the outcome (VAS or ODI score) is modeled as:

*outcome_it_* = *β*_0_ + *f*·(*time_it_*) + *β*_1_·*covariate_i_* + … + *β_n_·covariate_i_* + *b*_0*i*_ + *b*_1*i*_·(*time_it_*) + *ϵ_it_*,

where *β*_0_: fixed intercept, representing the expected baseline value of outcome for the population average when all predictors are at their reference levels; *f*·(*time_it_*): nonlinear fixed effect of time, modeled using natural cubic splines (capturing its nonlinear nature by Figure 2 and Figure 3); *β*_1_, …, *β_n_*: fixed effects for *n* covariates; *b*_0*i*_: random intercept for patient *i*, representing their deviation from the population-level intercept (*β*_0_); *b*_1*i*_: random slope for time for patient *i*, representing their deviation from the population-level trajectory of time; *ϵ_it_*: residual error for patient *i* at time *t*, assumed to follow *N*(0, *σ*^2^*)*.

**Level 2: Between-patient model**

The random effects (*b*_0ib_ and *b*_1ib_) are modeled at the patient level as:

*b*_0*i*_ ∼ *N*(0, *σ_b_*_0_^2^),

*b*_1*i*_ ∼ *N*(0, *σ_b_*_1_^2^),

*Cov*(*b*_0*i*_,*b*_1*i*_) = *σ_b_*_0*b*1,_

where: *σ_b_*_0_^2^: variance of the random intercepts, representing variability in baseline outcome levels across patients; *σ_b_*_1_^2^: variance of the random slopes for time, representing variability in the effect of time across patients; *σ_b_*_0*b*1_: covariance between random intercepts and random slopes, capturing the relationship between baseline outcome levels and the rate of change over time.
